# Chitosan–sEPDM and Melatonin–Chitosan–sEPDM Composite Membranes for Melatonin Transport and Release

**DOI:** 10.3390/membranes13030282

**Published:** 2023-02-27

**Authors:** Florentina Mihaela Păncescu, Abbas Abdul Kadhim Klaif Rikabi, Ovidiu Cristian Oprea, Alexandra Raluca Grosu, Aurelia Cristina Nechifor, Vlad-Alexandru Grosu, Szidonia-Katalin Tanczos, Florina Dumitru, Gheorghe Nechifor, Simona Gabriela Bungău

**Affiliations:** 1 Analytical Chemistry and Environmental Engineering Department, University Politehnica of Bucharest, 011061 Bucharest, Romania; 2 Al–Mussaib Technical College, Al–Furat Al–Awsat Technical University (ATU), Babylon–Najaf Street, Kufa 54003, Iraq; 3 Department of Inorganic Chemistry, Physical Chemistry and Electrochemistry, University Politehnica of Bucharest, 011061 Bucharest, Romania; 4 Department of Electronic Technology and Reliability, Faculty of Electronics, Telecommunications and Information Technology, University Politehnica of Bucharest, 061071 Bucharest, Romania; 5 Department of Bioengineering, University Sapientia of Miercurea-Ciuc, 500104 Miercurea-Ciuc, Romania; 6 Department of Pharmacy, Faculty of Medicine and Pharmacy, University of Oradea, 410028 Oradea, Romania

**Keywords:** melatonin, composite membranes, chitosan, sEPDM, melatonin transport and release

## Abstract

Melatonin is the hormone that focuses the attention of the researchers in the medical, pharmaceutical, materials, and membranes fields due to its multiple biomedical implications. The variety of techniques and methods for the controlled release of melatonin is linked to the multitude of applications, among which sports medicine occupies a special place. This paper presents the preparation and characterization of composite membranes based on chitosan (Chi) and sulfonated ethylene-propylene-diene terpolymer (sEPDM). The membranes were obtained by controlled vacuum evaporation from an 8% sEPDM solution in toluene (*w/w*), in which chitosan was dispersed in an ultrasonic field (sEPDM:Chi = 1:1, *w/w*). For the comparative evaluation of the membranes’ performances, a melatonin-chitosan-sulfonated ethylene-propylene-diene terpolymer (Mel:Chi:sEPDM = 0.5:0.5:1.0, *w/w/w*) test membrane was made. The prepared membranes were morphologically and structurally characterized by scanning electron microscopy (SEM), Fourier transform infrared spectroscopy (FTIR), energy-dispersive spectroscopy analysis (EDAX), thermal analysis (TG, DSC), thermal analysis coupled with chromatography and infrared analysis, and contact angle measurements, but also from the point of view of performance in the process of transport and release of melatonin in dedicated environments (aqueous solutions with controlled pH and salinity). The prepared membranes can release melatonin in amounts between 0.4 mg/cm^2^·per day (sEPDM), 1.6 mg/ cm^2^·per day (Chi/sEPDM), and 1.25 mg/cm^2^·per day (Mel/Chi/SEPDM).

## 1. Introduction

The biomedical implications of melatonin, the hormone secreted by the pineal gland, are so diverse and of particular importance that researchers have devoted extensive studies to it [1,2,3].

One of the current medical concerns is ensuring the daily amount of melatonin in the body, because the secretion of the pineal gland can be affected by various dysfunctions of the human body [4,5]. Even getting older is a problem in reducing the amount of melatonin generated in the body [6].

Starting with the circadian cycle [7], melatonin is involved in a series of biochemical processes related to: reducing oxidative stress [8], increasing aerobic performances [9], tissue repair [10], muscle adaptation and skeletal muscle capacity [11], sleep and neuroprotection [12], blood pressure [13], metabolic diseases [14], cancer [15], rheumatic diseases [16], trauma and accidents [17], stimulation of cartilage matrix development [18], cardiovascular diseases [19], arthritis [20], and depression [21]. In order to have an overview of all of melatonin’s connections with the body’s health level, one can imagine a spiral scheme of its effects on some diseases or conditions of the body (Figure S1, see supplementary material).

The diversity of melatonin’s implications in human body conditions has led to a variety of administration methods. The oral one predominates in current treatments because melatonin is easily adsorbed and desorbed from the digestive system, allowing the use of multiple materials and forms of conditioning [22,23,24].

However, there are more and more cases: trauma following various accidents [25], contusions [26], dislocation of bones from the joints [27], broken or displaced teeth in contact sports or that use special equipment or devices [28], blows to the head and spine [29], fractures and various accidents in the practice of motorized sports [30], which require the application and delivery of melatonin locally, alone or accompanying other drugs or pharmaceutical preparations [31].

**Figure 1:**
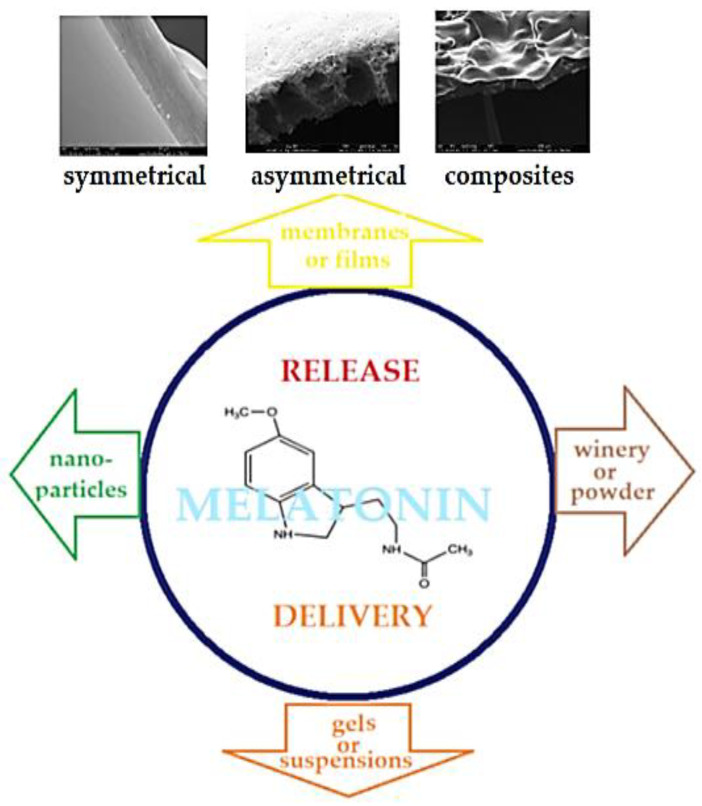
Schematic presentation of interest for chemical species’ (melatonin) release or delivery techniques.

The representative ways of delivery, controlled release of the various chemical species of interest, many of which have been studied for melatonin as well, are suggested in Figure 1 [32,33,34,35,36,37,38,39,40,41,42,43,44].

Among the recent applications of melatonin with significant results are those in sports medicine, while among the methods of controlled release, the attention given to the involvement of chitosan in various formulations can be highlighted [35].

Chitosan ensures a controlled release of melatonin, especially through ingestion, but for applications in sports medicine, orthopaedics, or dentistry, a reduction in the solubility of this biopolymer and an improvement in physical stability are necessary [36]. By embedding in various organic or inorganic nanoparticles, films, and membranes from biodegradable polymers, it is possible to reduce the solubility of chitosan [37].

Composite membranes are effective means both for the transport and the delivery or controlled released of melatonin [38], which justifies the expansion research in the field [39].

Studies related to improving the performance of fuel cell membranes have shown that an effective means of chitosan reticulation can be performed with ionophores with sulfonic groups of the polyether–ether sulfonated ketone type [40].

The study presented in this paper refers to the preparation and characterization of a composite membrane based on chitosan (Chi) and sulfonated ethylene-propylene-diene terpolymer (sEPDM) and its controlled melatonin release performance in synthetic aqueous solutions. The study addressed the transport and release of melatonin through the composite membrane (Chi-sEPDM) compared to the integral membrane from sEPDM. On the other hand, the results of the controlled release through the composite membrane (Chi-sEPDM) were compared to the parallel measurements of melatonin release from a melatonin/chitosan/sulfonated ethylene-propylene-diene terpolymer (Mel-Chi-sEPDM) test composite membrane. The operational parameters of the melatonin transport and release experiments from a saturated source phase (SP) or from the test membrane were the pH and salinity (NaCl) of some synthetic receiving aqueous solutions (RP).

## 2. Materials and Methods

### 2.1. Reagents and Materials

All reagents and organic compounds used in the presented work were of analytical grade. They were purchased from Merck (Merck KGaA, Darmstadt, Germany): hydrochloric acid, sodium chloride, and sodium hydroxide.

Melatonin (Mel-Sigma-Aldrich, Merck KGaA, Darmstadt, Germany) and the basic polymeric materials for making the membranes, i.e., chitosan (Chi) (Sigma-Aldrich Chemie GmbH, Steinheim, Germany) and sulfonated ethylene-propylene-diene terpolymer (sEPDM), were recently used in our research group for ionic and molecular separations [41]. Their main characteristics are given in Table 1.

The purified water characterized by 18.2 μS/cm conductivity was obtained with a RO Millipore system (MilliQ^®^ Direct 8 RO Water Purification System, Merck, Darmstadt, Germany) [42].

### 2.2. Methods and Procedures

#### 2.2.1. Obtaining the Composite Membranes Based on SEPDM

Obtaining the membranes from sEPDM (M1), chitosan–sEPDM (M2), and melatonin–chitosan–sEPDM (M3) was carried out by phase inversion method [41] and by controlled evaporation technique [43].

**Figure 2:**
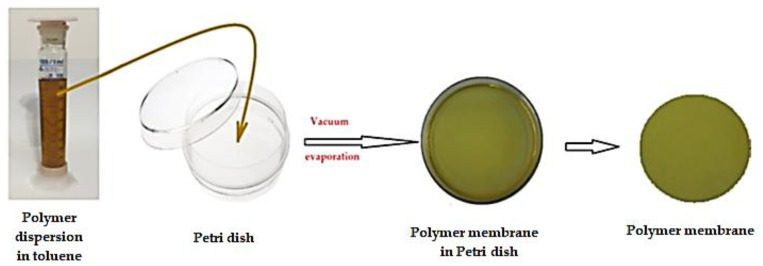
Schematic representation of the procedure for obtaining sEPDM based membranes.

The polymer solution (sEPDM 8%, *w/w*) in toluene is introduced in a Petri dish and evaporated in a vacuum oven at a temperature of 60 °C, thus obtaining the polymer membrane from sEPDM (M1) (Figure 2).

To obtain the chitosan with sEPDM (M2) composite membrane, a dispersion of chitosan in toluene solution of sEPDM was made, by introducing 1 g of chitosan in 12.5 g of sEPDM solution (8% in toluene) so that the mass ratio of the two polymers is 1:1. To disperse the chitosan, the glass bottle was placed in an ultrasonic bath (Elmasonic S, Elma Schmidbauer GmbH, Singen, Germany) for three hours, observing the complete dispersion. The dispersion is introduced into a Petri dish and evaporated under controlled conditions at 60 °C, under vacuum.

The preparation of the melatonin–chitosan–sEPDM composite membrane (M3) is carried out by using a mixture of melatonin and chitosan powders (0.5 g:0.5 g), which is homogenized in a dry way, for two hours, at 100 rpm, in a mill with spherical ceramic bodies (Retsch PM100 mill form Viola Schimadzu, Bucharest, Romania). The obtained powder is dispersed in 12.5 g toluene solution of sEPDM (8%, *w/w*), in the Elmasonic S ultrasonic bath, for three hours, obtaining a dispersion in toluene with a melatonin:chitosan:sEPDM ratio = 0.5 g:0.5 g:1.0 g. The dispersion is then introduced into a Petri dish and evaporated under controlled conditions at 60 °C, under vacuum.

In all three cases, the complete evaporation of toluene was followed, by bringing the STERIPLAN ROTH Petri bottles to constant mass.

The membranes were cut into 1.2 cm and 3.3 cm diameter disks, intended for morphological, structural, transport, and release characterizations. The prepared membranes were morphologically and structurally characterized by scanning electron microscopy (SEM), Fourier transform infrared spectroscopy (FTIR), energy-dispersive spectroscopy analysis (EDAX), thermal analysis (TG, DSC), thermal analysis coupled with chromatography and analysis in infrared, and contact angle measurements, but also from the point of view of performance in the process of transport and release of melatonin in dedicated environments (aqueous solutions with controlled pH and salinity).

#### 2.2.2. Transport and Release of the Melatonin through/from Composite Membranes Based on SEPDM

##### Transport Performance of the Obtained Membranes

**Figure 3:**
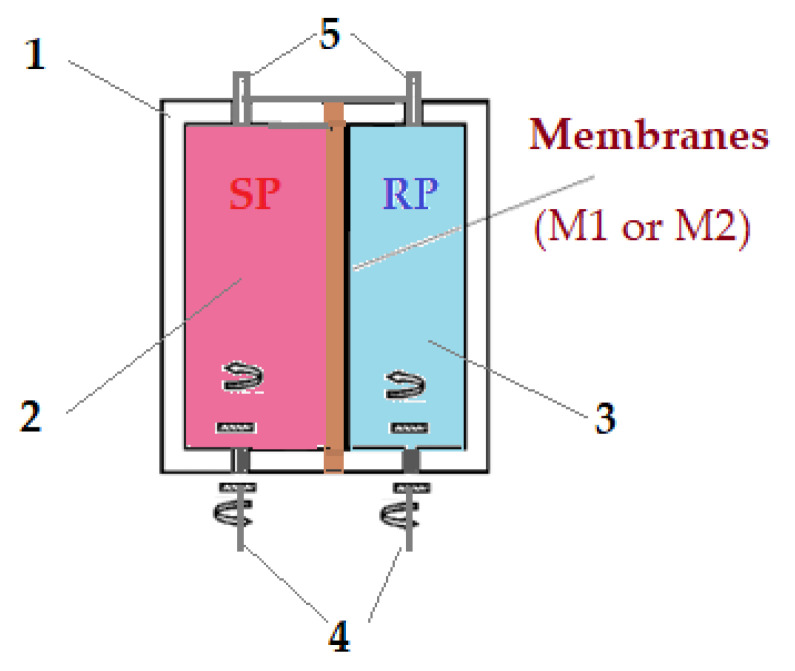
Schematic representation of pertraction installation for the melatonin transport through prepared membranes (M1 and M2): 1-pertraction module; 2-source compartment; 3-receiving compartment; 4-magnetic stirrer system; 5-pipes; source phase (SP – 2g/L melatonin neutral aqueous solution); receiving phase (RP – pH and salinity-controlled solution).

To determine the transport performances of the prepared membranes (M1 or M2), a permeation module (Figure 3) with two compartments separated by a disk with a free membrane diameter of 3.3 cm was used [44]. Both compartments have a stirring magnetic bar (50 rpm) placed at the base. In one compartment, a 100 mL source solution of 2.0 g/L melatonin in ultra-pure water is introduced (the source phase, SP), and, in the second compartment, 100 mL synthetic receiving solutions of imposed pH made with hydrochloric acid or sodium hydroxide are introduced, in a range close to the biological pH (the receiving phase, RP).

In another set of experiments, 1–5% NaCl salinity solutions in ultra-pure water were used as the receiving phase. The experiments were carried out in five identical bipartite modules, with a volume of 100 mL melatonin solution and an imposed pH or salinity solution of the same volume, so that the results can be averaged. The five membranes, dedicated to each set of tests, were kept for 48 h in the 2 g/L melatonin solution and wiped by gentle pressing between two filter paper discs (Whatman^®^ Filter Paper, Merck KGaA, Darmstadt, Germany) and then were fixed by silicone rubber gaskets in the permeation modules. The spectrophotometric analyses were performed daily, at two wavelengths, 278 nm, and 285 nm, for ten days, collecting 1.0 mL of solution from the source phase. The analyses were performed on two different spectrometers, by the same operator and repeated by an independent operator. The analysis laboratory works and respects the specific recommendations and guidelines of EURACHEM [45]. The validation of the analysis method was carried out by a fast and sensitive electrochemical method, developed and reported previously [46].

##### Release Performance of the Obtained Membranes

The controlled release experiments were carried out according to a previously described procedure [47].

**Figure 4:**
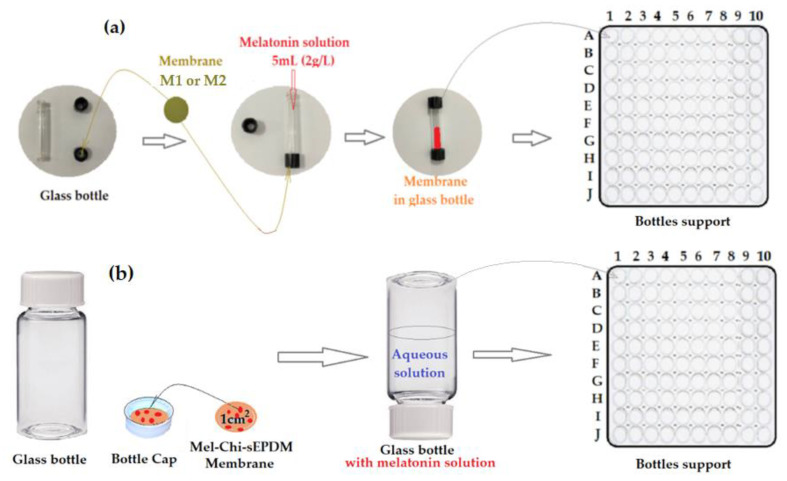
Release schematic procedure “through” prepared membranes (M1 and M2) (**a**); and release schematic procedure “from” composite membrane (M3) (**b**).

In this study, two types of melatonin release are experimented:

1. The release of melatonin from a saturated solution “through” membranes prepared in synthetic solutions of controlled pH and salinity (Figure 4a);
2. The release of melatonin “from a” test composite membrane (Mel-Chi-sEPDM, M3) in synthetic solutions of controlled pH and salinity (Figure 4b).

In the first case, the membrane discs were placed in the lids of 10 cm^3^ glass bottles. Then, 5.0 mL of controlled 2 g/L melatonin aqueous solution was introduced into the glass bottles, and the perforated bottle cap was sealed with a membrane and placed with the cap down in a cup in which 100 bottles could be inserted simultaneously (Figure 4a). The entire assembly is placed in a vessel (10.0 L, simulating a high, quasi-constant dilution, and to maintain the concentration gradient for melatonin), with controlled pH or salinity solution, which is recirculated with a constant flow rate (200 mL/min). Seven bottles were retrieved daily for analysis so that the results of the melatonin analysis could be averaged, and three bottles were stored as control samples.

In the second case, the composite membranes (Mel-Chi-sEPDM, M3) are placed in the lid of the 100 bottles containing 5.0 mL synthetic solution of imposed pH and salinity, and melatonin from the membranes is released in contact with them (through overturning) (Figure 4b).

As in the previous case, seven bottles were retrieved daily for analysis so that the results of the melatonin analysis could be averaged, and three bottles were stored as control samples.

The validation of the results was performed periodically by electrochemical and/or UV–Vis methods at an independent laboratory [47,48].

The flows of the melatonin derivatives from the source phase were determined at specific time intervals, using the relation (1) [49]:

(1) $$J=\frac{M}{S\cdot \Delta t}\;(\mathrm{mg}/(m^{2}\;s))\;\mathrm{or}\;\mathrm{mol}/(m^{2}\;s))$$

*M* being the permeate mass (g or mol), *S* being the effective surface of the membrane (m^2^), and Δ*t* the time interval (s).

The release (or pertraction) efficiency RE% (or PE%) for the melatonin derivatives was calculated as follows [50], based on melatonin solution concentration:

(2) $$RE\;or\;PE\left(\%\right)=\frac{\left(c_{0}-c_{f}\right)}{c_{0}}\cdot 100$$

*c_f_* being the final concentration of the solute (melatonin) and *c*_0_ the initial concentration of solute (melatonin).

The same release efficiency can also be obtained based directly upon the absorbance of the considered solutions (melatonin) [51], as in (3):

(3) $$RE\;or\;PE\left(\%\right)=\frac{\left(A_{0}-A_{s}\right)}{A_{0}}\cdot 100$$

*A*_0_ being the initial absorbance of the sample melatonin solution and *A_s_* the current absorbance of the sample.

### 2.3. Equipment

The surface and cross-section characteristics of the membranes were determined with a scanning electron microscopy (SEM) equipped with a probe for energy dispersive spectroscopy analysis (EDX). A Hitachi S4500 system was used (Hitachi High-Technologies Europe GmbH, Krefeld, Germany) [52].

Thermal analysis (TG-DSC) was performed with a STA 449C Jupiter apparatus, from Netzsch (NETZSCH-Gerätebau GmbH, Selb, Germany). Each sample weighed approximatively 10 mg. The samples were placed in an open alumina crucible and heated up to 900 °C with 10 K∙min^−1^ rate, under flow of 50 mL∙min^−1^ dried air. As reference, we used an empty alumina crucible. The evolved gases were analysed with a FTIR Tensor 27 from Bruker (Bruker Co., Ettlingen, Germany), equipped with a thermostat gas cell [53].

FTIR 2D maps were recorded with a Nicolet iS50R FTIR microscope (Thermo Fisher Scientific Inc., Waltham, MA, USA), with a deuterated triglycine sulphate (DTGS) detector, in the wavenumber range 4000–600 cm^−1^. The FTIR 2D maps were used to obtain information about the spatial distribution of the components [54].

Determination and monitoring of pH and salinity for every stock solution was achieved using a conductance cell or combined selective electrode (HI 4107, Hanna Instruments Ltd., Leighton Buzzard, UK) and a multi-parameter system (HI 5522, Hanna Instruments Ltd., Leighton Buzzard, UK) [55].

The UV–Vis spectra of the melatonin samples were recorded for a wavelength ranging from 200 to 800 nm, at room temperature, using 10 mm quartz cells on CamSpec M550 spectrometer (Spectronic CamSpec Ltd., Leeds, UK), and, for the daily determinations, two wavelengths were chosen, 278 nm and 285 nm [56,57].

Additionally, the UV–Vis validation analysis of the melatonin solutions was performed on dual-beam UV equipment—Varian Cary 50 (Agilent Technologies Inc., Santa Clara, CA, USA)—at a resolution of 1 nm, spectral bandwidth of 1.5 nm, and a scan rate of 300 nm/s [57].

Contact angle measurements for the considered spheres materials (with distilled water or melatonin derivatives solution) [58] were carried out with a horizontal microscope with video camera (Viola–Shimadzu, Bucharest, Romania).

## 3. Results and Discussion

The controlled release of pharmaceutical preparations is an important aspect that doctors take into account both when prescribing drug doses and when administering food supplements [59,60,61,62].

In the case of melatonin, the possibility of oral administration allows its inclusion in powdery materials, tablets, or cassettes, which, by ingestion, ensure the release of controlled amounts in the body [63]. If a localized administration is desired (injuries, trauma, areas of the oral cavity), as is the case in sports accidents, creams, gels, patches, or films (membranes) can be used [64,65,66,67,68,69].

In the present study, the controlled transport or release of melatonin through a chitosan (Chi)-sulfonated ethylene-propylene-diene terpolymer (Chi-sEPDM) composite membrane, with possible applications in sports medicine, were considered. The results of the controlled release through the composite membrane (Chi-sEPDM) were compared to the parallel measurements of melatonin release from a melatonin-chitosan-sulfonated ethylene-propylene-diene terpolymer composite test membrane (Mel-Chi-sEPDM).

The membrane prepared by controlled evaporation from a chitosan or a melatonin–chitosan dispersion in sEPDM solution in toluene was characterized morphologically and structurally by scanning electron microscopy (SEM), Fourier transform infrared spectroscopy (FTIR), energy-dispersive spectroscopy analysis (EDAX), thermal analysis (TG, DSC), thermal analysis coupled with chromatography and infrared analysis, but also from the point of view of melatonin transport to solutions of controlled pH and salinity.

### 3.1. Morphological and Structural Membrane Characteristics

The obtained membranes have the macroscopic characteristics illustrated in Table 2: the thickness, the general aspects (photo), low resolution scanning electron microscopy, and the contact angle with distilled water. The membranes have very different appearance, roughness, morphology, and wetting characteristics, which can be found in the performance of transport and/or release of melatonin in aqueous solutions of variable pH and salinity. Although the thickness of the three membranes is relatively similar, in the micrometric determinations, it is still affected by the very different roughness [70]. The hydrophilicity of the membranes increases sharply in the series M1 < M2 < M3, both through the influence of the composition and the roughness found in low resolution scanning electron microscopy (Table 2).

#### 3.1.1. Scanning Electron Microscopy (SEM)

The membrane samples based on sulfonated ethylene-propylene-diene terpolymer (sEPDM) (M1), chitosan-sulfonated ethylene-propylene-diene terpolymer (Chi-sEPDM) (M2), and melatonin-chitosan-sulfonated ethylene-propylene-diene terpolymer (Mel-Chi-sEPDM) (M3) with a size of 10 cm^2^, were fractured in liquid nitrogen and metallized with a superficial layer of gold, to be able to examine the section of the membranes (scanning electron microscopy, SEM) and the elemental distribution on the surface (energy-dispersive spectroscopy analysis, EDAX), analyses available on a Hitachi S4500 system.

**Figure 5:**
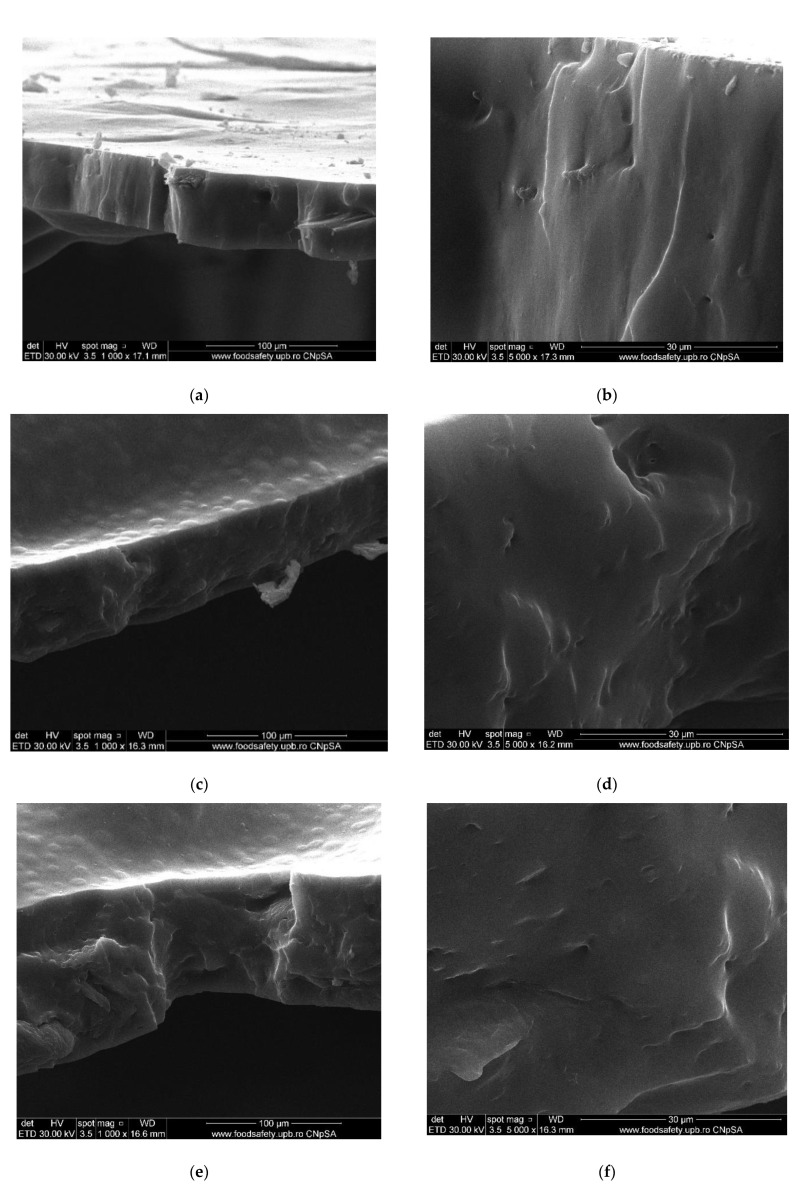
Scanning electron microscopy (SEM) images for: sulfonated ethylene-propylene-diene terpolymer (sEPDM) (M1)—(**a**,**c**,**e**); chitosan (Chi)-sulfonated ethylene-propylene-diene terpolymer (Chi-sEPDM) (M2)—(**b**,**d**,**f**).

**Figure 6:**
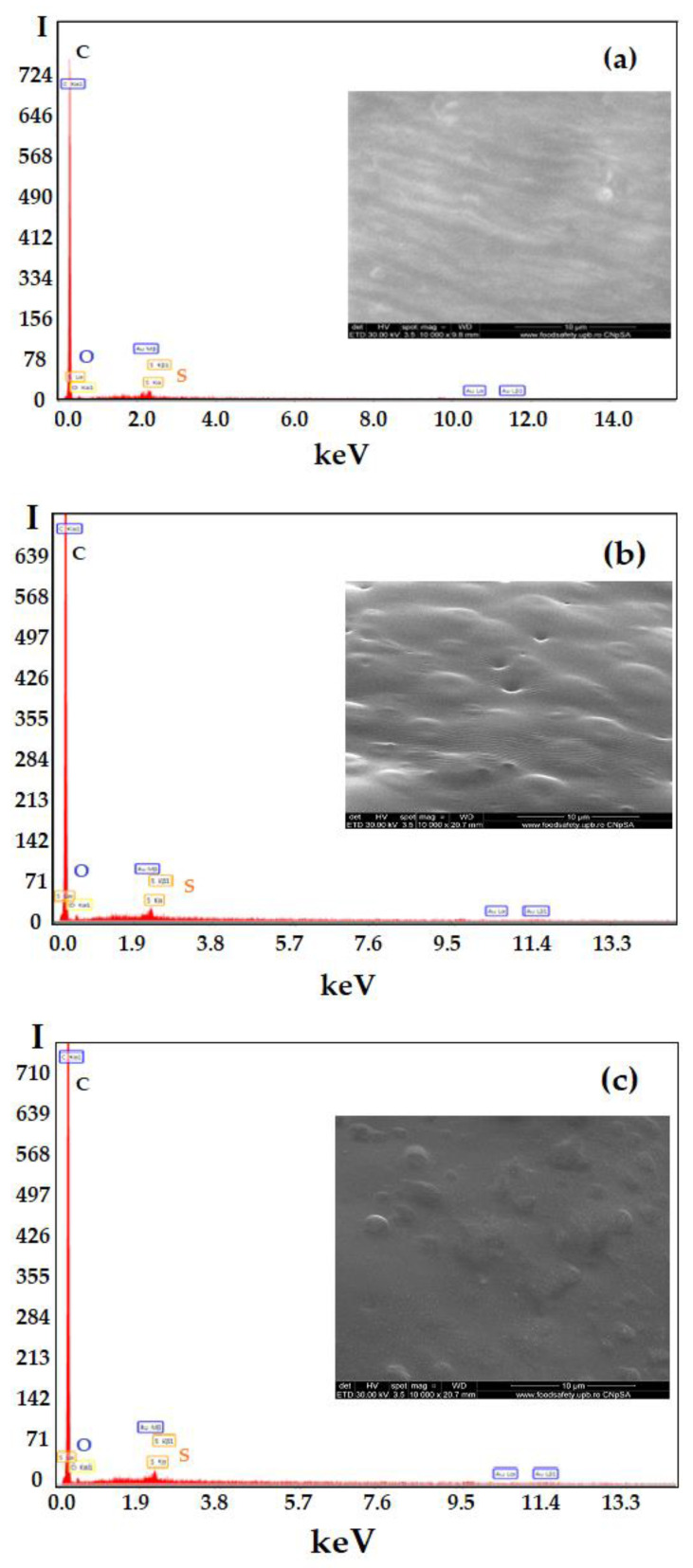
Energy-dispersive spectroscopy analysis (EDAX) diagram for: sulfonated ethylene-propylene-diene terpolymer (sEPDM) (M1) (**a**); chitosan (Chi)–sulfonated ethylene-propylene-diene terpolymer (Chi-sEPDM) (M2) (**b**), melatonin/chitosan (Chi)–sulfonated ethylene-propylene-diene terpolymer (Mel-Chi-sEPDM) (M3) (**c**).

Figure 5 shows the images obtained for the three membranes (M1, M2 and M3), at a magnification of ×1000 for the cross-section (Figure 5a,c,e), and the cross-section details ×5000 (Figure 5b,d,f). Figure 6 illustrates the elemental composition.

The uniformity of the sEPDM membrane surface (M1, Figure 5a) is specific to an integral membrane obtained by phase inversion through evaporation, both on the surface and in the section. The electrostatic charge causes the adhesion of several polymer fragments on the surface, and isolated crystallites are observed in the section (Figure 5a,b).

The images of the two composite membranes (M2 and M3) (Figure 5b–f) highlight the heterogenous nature, the micro-aggregates of chitosan or melatonin being clearly observed especially on the surfaces (Figure 5c,d) but also in sections (Figure 5d,f). These superficial agglomerations amplify to a microscopic level the roughness of the two composite membranes, also observed in the assembly presented in Table 2.

The elemental analysis on the surface (EDAX) allows highlighting of carbon (C) elements, the majority, but also oxygen (O) and sulphur (S) on all membranes: the sulfonated ethylene-propylene-diene terpolymer (sEPDM) membrane (M1) (Figure 6a) and in the chitosan-sulfonated ethylene-propylene-diene terpolymer (Chi-sEPDM) composite membrane (M2) (Figure 5b), and melatonin-chitosan-sulfonated ethylene-propylene-diene terpolymer (Mel-Chi-sEPDM) composite membrane (M3) (Figure 5c).

The surface elemental concentration is slightly different for all membranes (Table 3). However, one observes a remarkable reduction with almost half of the sulphur concentration and with a third of the oxygen concentration in the case of the Chi-sEPDM membrane (M2), compared to the melatonin-chitosan-sulfonated ethylene-propylene-diene terpolymer (Mel-Chi-sEPDM) composite membrane (M3) in the examined areas.

Although the difference in elemental surface composition is relatively small, the hydrophilicity of the membranes’ surfaces changes dramatically (Table 2): the composite membrane (M3) being much more hydrophilic (θ = 35°), comparatively with the composite membrane (M2) being more hydrophilic (θ = 45°), and much more than the sEPDM membrane (M1, θ = 75°). This observation shows, for the present case, that the surface roughness is decisive for the hydrophilicity of the membranes.

#### 3.1.2. Fourier Transform InfraRed Spectroscopy (FTIR) Membrane Characteristics

The data obtained from the elemental analysis (EDAX) required a study in the infrared domain both spectrally (FTIR) and by interference reflection microscopy (IRM), which would provide more structural information and surface composition.

**Figure 7:**
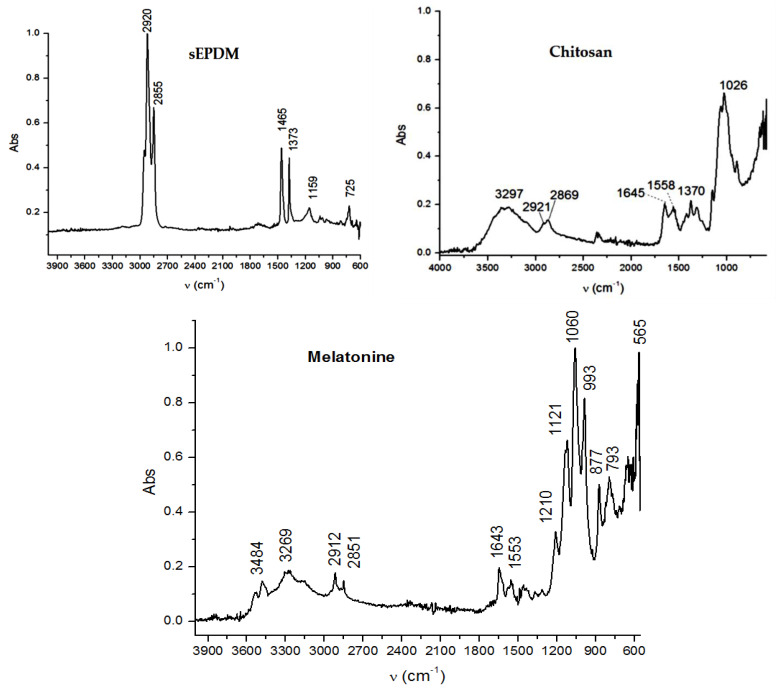
Fourier transform infrared spectra for: sulfonated ethylene-propylene-diene terpolymer (sEPDM); chitosan (Chi), and melatonin (Mel).

Figure 6 shows the spectra of the base materials: sulfonated ethylene-propylene-diene terpolymer (sEPDM), chitosan (Chi), and melatonin (Mel) (Figure 7).

The spectra obtained were used to select the wave numbers for which the infrared microscopy map (FTIR, 2D) was made, for the three membranes obtained.

Most of the specific wave numbers of the three materials are located in very close areas and therefore cannot be used as safe specific values for the FTIR microscopy study. It should also be emphasized that the sEPDM film subjected to FTIR analysis was obtained from toluene solution, which did not favour the highlighting of hydrogen bonds of the sulfonic group (Figure 7). The examined chitosan and melatonin were presented as a powder, and the obtained spectra are compatible with the literature data.

**Figure 8:**
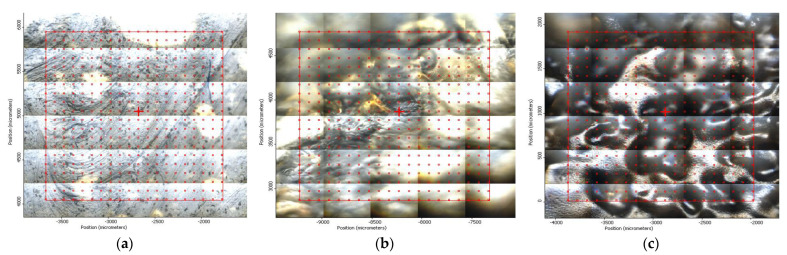
Video images for; (**a**) sulfonated ethylene-propylene-diene terpolymer (sEPDM) membrane (M1); (**b**) chitosan-sulfonated ethylene-propylene-diene terpolymer (Chi-sEPDM) membrane (M2); and (**c**) melatonin-chitosan-sulfonated ethylene-propylene-diene terpolymer (Mel-Chi-sEPDM) membrane (M3).

In another train of thought, the three materials used to obtain the composite membrane interact. Thus, the sulfonic groups in sEPDM give a neutralization reaction with primary amino groups from chitosan and secondary amino groups from melatonin, but there are also other possible interactions such as hydrogen bonds, ionic bonds, and hydrophobic bonds (Table 1). Figure 8 shows the images of the selected areas (M1 in Figure 8a, M2 in Figure 8b, and M3 in Figure 8c), and Table 4 shows FTIR 2D maps, at randomly selected wave numbers, but targeting each representative range of the spectra: 3345 cm^−1^, 1385 cm^−1^, 1050 cm^−1^, and 728 cm^−1^.

**Figure 9:**
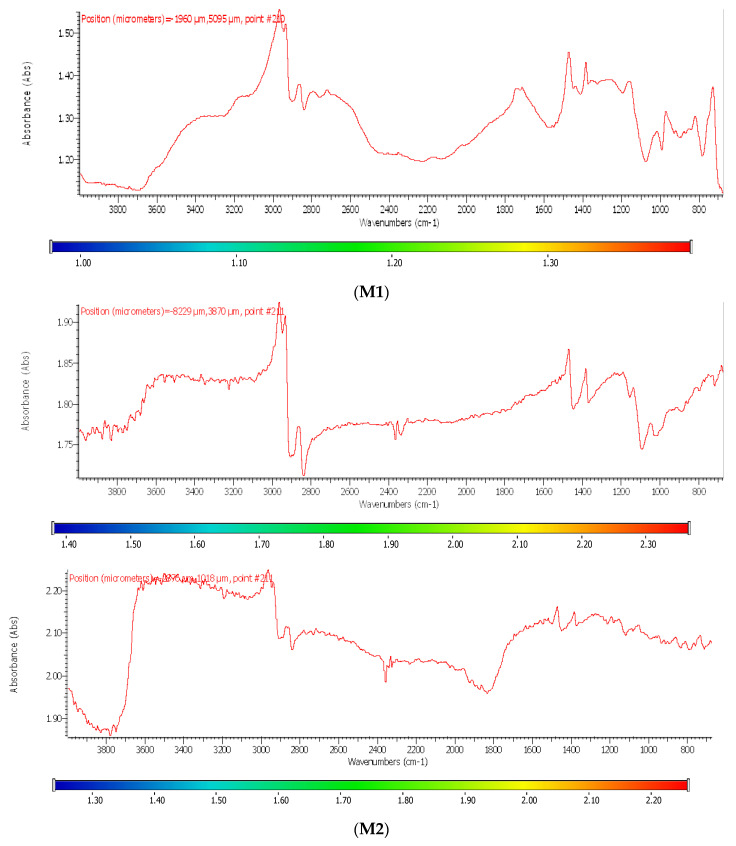

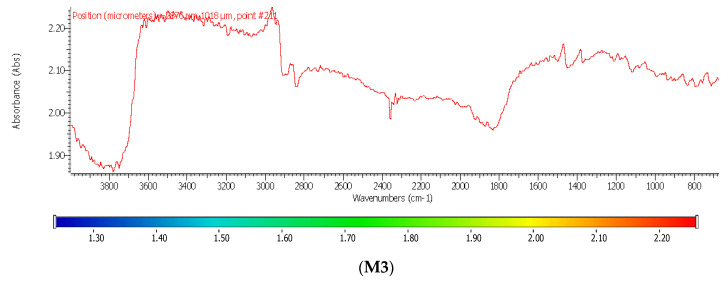
Infrared associated spectrum and colour scales for sulfonated ethylene-propylene-diene terpolymer (sEPDM) membrane (**M1**); chitosan-sulfonated ethylene-propylene-diene terpolymer (Chi-sEPDM) membrane (**M2**), and melatonin-chitosan-sulfonated ethylene-propylene-diene terpolymer (Mel-Chi-sEPDM) membrane (**M3**).

The associated spectra and the colour scale used are shown in Figure 9, showing significant differences that can largely justify the differences in hydrophilicity presented by the prepared membranes (Table 1 and Table 4).

The HD-IR obtained maps show a relatively uniform, regular, and repeatable distribution of the surface of the obtained membranes, especially for the sulfonated ethylene-propylene-diene terpolymer (sEPDM) membrane (M1). The composite chitosan-sulfonated ethylene-propylene-diene terpolymer (Chi-sEPDM) membrane (M2), and melatonin-chitosan-sulfonated ethylene-propylene-diene terpolymer (Mel-Chi-sEPDM) membrane (M3) show areas that, with the greatest probability, are due to the agglomeration of chitosan or melatonin–chitosan (upper left corner of the images), being more obvious for a wave number of 3345 cm^−1^, but also present for all other wave numbers (see Table 4). This agglomeration was also highlighted in the scanning electron microscopy details (Figure 5c,e).

#### 3.1.3. Thermal Characteristics of the Prepared Membranes

The complex thermal analysis had both the role of highlighting the thermal behaviour of the membranes at relatively low temperatures (up to 300 °C) and their composition through gas chromatographic analysis coupled with infrared spectrometry of combustion gases (up to 800 °C).

**Figure 10:**
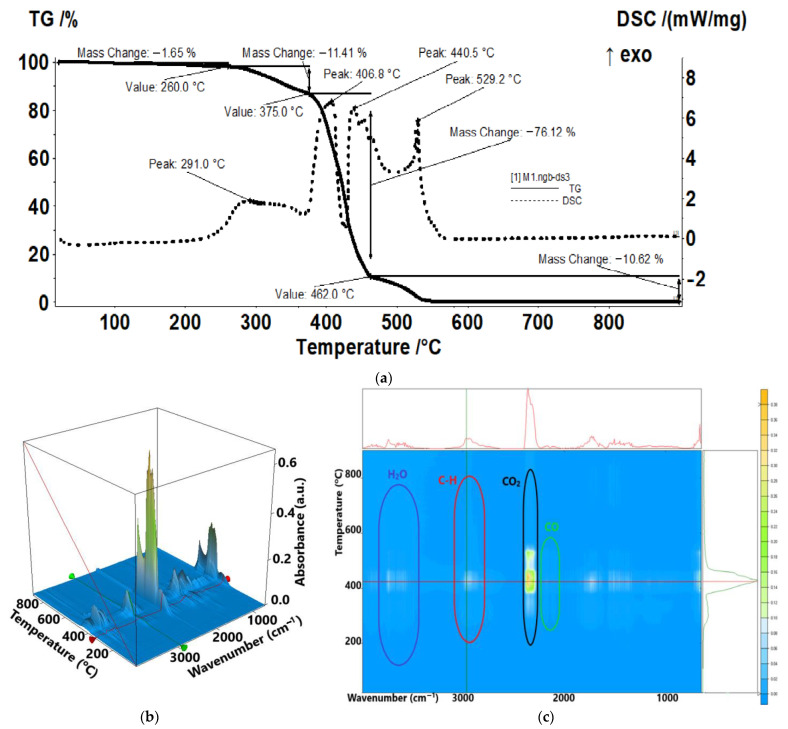
Thermal characteristics of the sulfonated ethylene-propylene-diene terpolymer (sEPDM) membrane (M1): (**a**) thermal diagram; (**b**) 3D complex analysis; (**c**) 2D complex analysis.

The sample M1 (Figure 10) can be considered stable up to 260 °C, losing only 1.65% of its mass, mainly residual solvent but also some sulphur is removed as SO_2_, as indicated by the FTIR analysis of the evolved gases. Between 260–375 °C, the sample is losing 11.41% of its mass, the process being accompanied by a broad exothermic effect with peak at 291 °C. The main degradation process takes place between 375–462 °C, when the sample is losing 76.12% of its mass (Figure 10a). The DSC curve indicates two strong exothermic effects, but the FTIR of the evolved gases indicates a quasi-continuous production of CO_2_, H_2_O, or hydrocarbon fragments, which means that any backbone breaking in smaller fragments is also accompanied by the combustion of those fragments (Figure 10a–c). The FTIR spectrum at 429 °C, in the middle of the strongest degradation process, indicates the evolving of H_2_O, CO_2_, and CO as combustion products, but also of saturated hydrocarbon fragments from pyrolysis of the polymer backbone and SO_2_. The residual carbonaceous mass is burned after 460 °C, the process being accompanied by a strong exothermic peak at 529.2 °C. The FTIR analysis of evolved gases at 529 °C indicates that the product is mainly CO_2_. It can be seen that some desulfurization processes also take place under 200 °C.

The traces for evolving SO_2_ (1367 cm^−1^) vs. temperature and traces for evolving CO_2_ (2355 cm^−1^) vs. temperature were detailed in the supplementary material (Figure S2, see supplementary material).

**Figure 11:**
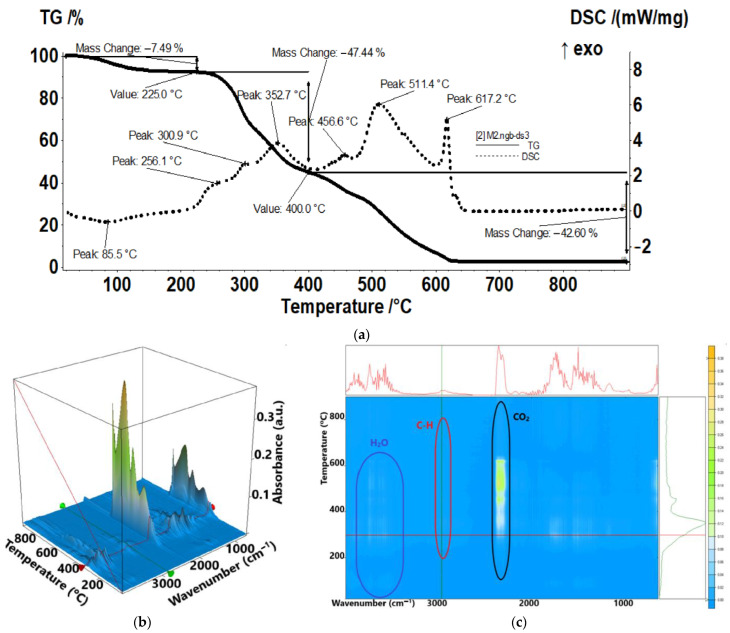
Thermal characteristics of the chitosan–sulfonated ethylene-propylene-diene terpolymer membranes (Chi-sEPDM) (M2): (**a**) thermal diagram, (**b**) 3D complex analysis; (**c**) 2D complex analysis.

The sample M2 (Figure 11) is losing 7.49% in the temperature interval RT–225 °C, the associated effect being weak and endothermic with minimum at 85.5 °C. The sample is losing some residual water molecules in this interval, but also the desulfurization processes start (Figure 11a), as indicated by the FTIR of the evolved gases and the traces for individual wavenumbers vs. temperature (Figure 11b). In the interval 225–400 °C, the sample begins to suffer an oxidative degradation, with multiple exothermic peaks visible on the DSC curve, partially overlapped. The FTIR of evolved gases allows identification of combustion products such as CO_2_, CO, and H_2_O, but also saturated hydrocarbons from polymer backbone fragmenting and SO_2_, indicating the complexity of the thermal degradation (Figure 10b,c). The majority of SO_2_ is evolving in this interval, with only minor peaks being identified on the compound trace after 400 °C (Figure 10a). The same can be stated for the saturated hydrocarbon fragments: after 400 °C, only a small peak is observable on the trace line (Figure 10c). After 400 °C, the sample suffers mostly oxidation processes, as indicated by the evolving of CO_2_ and H_2_O in larger quantities, culminating with the burning of the residual carbonaceous mass, which is accompanied by the strong and sharp exothermic effect from 617.2 °C. The recorded mass loss after 400 °C is 42.60%.

The traces for evolving SO_2_ (1367 cm^−1^), CO_2_ (2355 cm^−1^), hydrocarbons (2964 cm^−1^), and H_2_O (3566 cm^−1^) vs. temperature were detailed in the supplementary material (Figure S3, see supplementary material).

**Figure 12:**
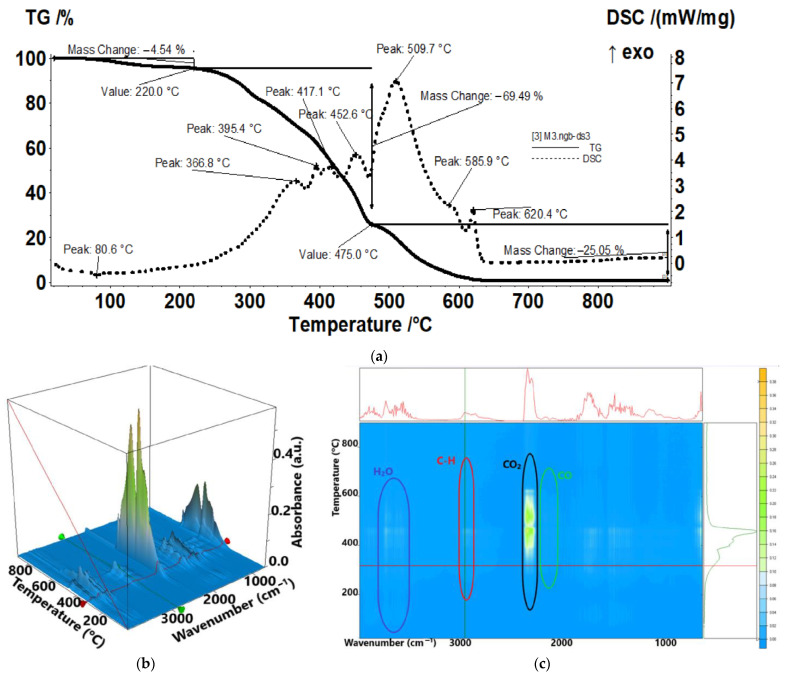
Thermal characteristics of the melatonin-chitosan-sulfonated ethylene-propylene-diene terpolymer membranes (Mel-Chi-sEPDM) (M3): (**a**) thermal diagram, (**b**) 3D complex analysis; (**c**) 2D complex analysis.

Sample M3 (Figure 12) is losing 4.54% up to 220 °C, mostly water molecules and some traces of SO_2_, as indicated by the FTIR spectra. The principal degradation processes take place between 220 °C and 475 °C, when a series of exothermic effects are seen on the DSC curve, indicating multiple oxidation reactions. The bulk of gaseous products CO, H_2_O, hydrocarbon fragments, and SO_2_ is eliminated in this interval. The recorded mass loss is 69.49%. After 475 °C, the residual carbonaceous mass is burned, the principal degradation product identified by FTIR being CO_2_.

The traces for evolving SO_2_ (1367 cm^−1^), CO_2_ (2355 cm^−1^), hydrocarbons (2964 cm^−1^), and H_2_O (3566 cm^−1^) vs. temperature were detailed in the supplementary materials (Figure S4, see supplementary material).

The results of complex thermal analysis, coupled with the gas chromatography and infrared spectrometry, provide the stability range of each prepared membrane but, above all, confirm their composition.

### 3.2. Transport and Release of the Melatonin through Prepared Membranes

A natural hormone, synthesized in the body, melatonin can be administered orally most of the time, and the main concern of the researchers was to find the most suitable methods of controlled release [71].

However, there are some specific aspects that make melatonin remain constantly in the attention of researchers in order to design new methods of delivery in the body:

- A universal dose of melatonin cannot be prescribed because each body has its own production [72];
- Age and health greatly affect the production of the pineal gland [73];
- The time of day is very important because the production of melatonin in the body is cyclical [74];
- In the case of accidents, especially those from various sports competitions, local administration is necessary (oral cavity, skin, bones, joints) [75,76].

All these considerations have encouraged experimental research on the transport and/or release of melatonin through composite membranes of chitosan-sulfonated ethylene-propylene-diene terpolymer membranes (Chi-sEPDM), and the melatonin-chitosan-sulfonated ethylene-propylene-diene terpolymer composite membranes (Mel-Chi-sEPDM), even if recently a mathematical model of the release of various active substances has been proposed [66].

In this study, the transport “through” the composite membrane obtained in a two-compartment membrane system and the release of melatonin “through” the composite membrane in an open system (the receiving solution is renewed) were followed.

For comparison, melatonin-chitosan-sulfonated ethylene-propylene-diene terpolymer (Mel-Chi-sEPDM) composite membrane was tested in a system for releasing melatonin “from inside” the membrane to a receiving phase consisting of synthetic solutions with imposed pH and salinity.

#### 3.2.1. Transport of Melatonin Transport through the Obtained Membranes (M1 and M2)

**Figure 13:**
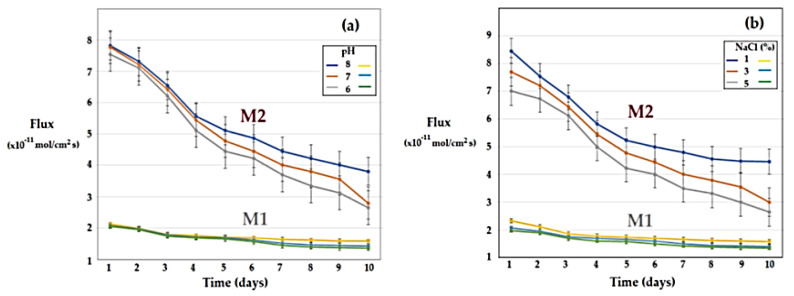
Time variation of melatonin flow through sulfonated ethylene-propylene-diene terpolymer (sEPDM) membranes (M1) and chitosan-sulfonated ethylene-propylene-diene terpolymer (Chi-sEPDM) membranes (M2), depending on pH (**a**) and salinity (**b**).

The melatonin transport experiments through sulfonated ethylene-propylene-diene terpolymer (sEPDM) membrane (M1) and chitosan-sulfonated ethylene-propylene-diene terpolymer (Chi-sEPDM) membrane (M2) were carried out from a 100 mL source phase, with a concentration of 2 g/L, and imposed pH (6.0, 7.0 and 8.0) or salinity (1.0%, 3.0% and 5.0% NaCl) 100 mL receiving phases. The compartments of the membrane system were constantly stirred. The results obtained (Figure 13) show that pH influences the transport, especially in the second part of the studied interval (Figure 13a), while sodium chloride has an effect on the transport from the beginning of the range, especially at lower concentrations (Figure 13b). The pronounced increase in salinity disfavours the transport, most probably by reducing the solubility of melatonin in the receiving phase.

**Figure 14:**
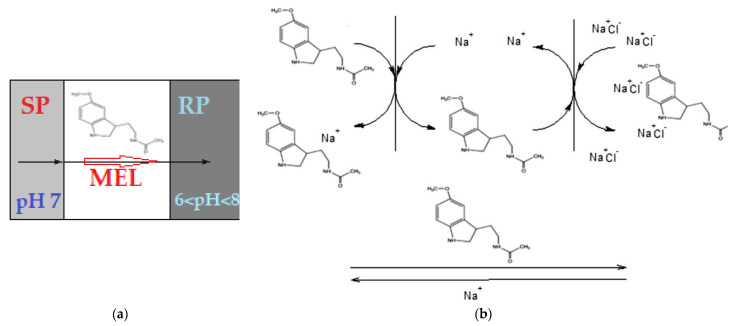
Transport schemes in the case of the receiving phase of controlled pH (**a**) or imposed salinity (**b**).

The transport of melatonin in the system with the receiving phase of variable pH is mainly determined by the difference in concentration between the aqueous phases, so by the solubility of melatonin in the aqueous phases of relatively close composition (Figure 13a). On the other hand, the transport of melatonin to the receiving phase of controlled salinity corresponds to a coupled transfer mechanism (Figure 13b), in which the melatonin transport from source phase to the receiving phase is coupled with the transport of sodium ions in the opposite direction (Figure 14).

#### 3.2.2. Release of Melatonin through the Obtained Membranes

The study of the controlled release of melatonin was carried out for a source phase of 5 mL, with a concentration of 2 g/L, and receiving phases of a much larger volume of 5 L with imposed pH (6, 7.0 and 8) or salinity (1%, 3% and 5% NaCl). Basically, a set of 100 vials containing the source phase is immersed in a vessel with 10.0 L of recirculated receiving solution, with a flow rate of 100 mL/min. Thus, it can be appreciated that the receiving phase will remain at the imposed pH and salinity. Ten of the vials from the set are taken out daily for analysis, for 10 days, seven of them for averaging the results and three to be sent for validation of the analyses.

**Figure 15:**
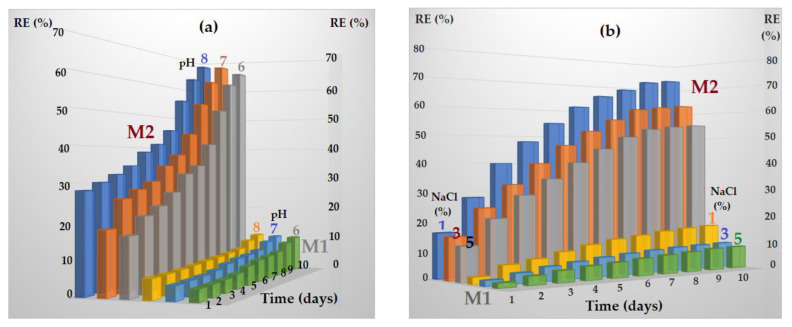
Time variation of melatonin cumulative release through sulfonated ethylene-propylene-diene terpolymer (sEPDM) membrane (M1) and chitosan-sulfonated ethylene-propylene-diene terpolymer (Chi-sEPDM) membrane (M2), depending on pH (**a**) and salinity (**b**).

The delivery results of melatonin for ten consecutive days through sulfonated ethylene-propylene-diene terpolymer (sEPDM) membrane (M1) and chitosan-sulfonated ethylene-propylene-diene terpolymer (Chi-sEPDM) membrane (M2) are presented in Figure 15.

The results obtained for the release of melatonin show that chitosan-sulfonated ethylene-propylene-diene terpolymer (Chi-sEPDM) membrane (M2) allows a faster transfer and in an amount that approaches the data from the literature, while sulfonated ethylene-propylene-diene terpolymer (sEPDM) membrane (M1) has a low but relatively constant release over time.

**Figure 16:**
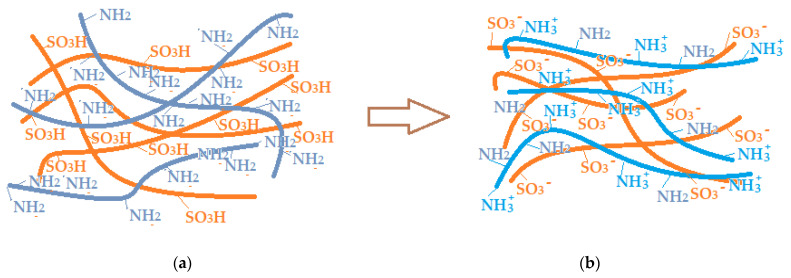
Schematic representation of the polymer mixture: before (**a**) and after (**b**) the formation of the chitosan–sulfonated ethylene-propylene-diene terpolymer membrane (Chi-sEPDM) (M2).

For the sulfonated ethylene-propylene-diene terpolymer (sEPDM) membrane (M1) that contains sulfonic reactive functional groups (SO_3_H), the interactions with melatonin during its transport and release are predictable, since at the working pH they are in sulfonate form (SO_3_−). In the case of chitosan-sulfonated ethylene-propylene-diene terpolymer (Chi-sEPDM) composite membrane (M2), the interactions with melatonin in transport and release are complex because at the working pH the amino groups can be free or ionized (ammonium) (Figure 16).

The ionic situation presented in Figure 16b is close to reality in the case of acidic pH, but at pH = 7 or higher, the ammonium groups will change to the amino form, and the membrane charge will be slightly negative (due to the sulfonate groups). All these considerations explain to a good extent not only the large difference between the hydrophilicity of the prepared membranes but also the influence of pH and salinity on the transport and release of melatonin.

The study of the sensitivity to pH variation for the case of chitosan-sulfonated ethylene-propylene-diene terpolymer (Chi-sEPDM) composite membrane (M2) requires a greater depth compared to the experiments carried out so far, by widening the range both towards strongly acidic and towards strongly basic environments.

#### 3.2.3. Melatonin Release from Melatonin-chitosan-sulfonated Ethylene-propylene-diene Terpolymer Composite Membranes (Mel-Chi-sEPDM, M3)

Melatonin-chitosan-sulfonated ethylene-propylene-diene terpolymer composite membranes (Mel-Chi-sEPDM, M3) were tested for the release of melatonin “from” their composition in synthetic solutions with pH and salinity similar to those of the previous experiments. The surface of the membranes in contact with 5.0 mL synthetic solution, from each of the 100 test bottles, is 1 cm^2^, and the mass of a membrane is 24.5 ± 0.5 mg, so the amount of melatonin in each membrane (available for release) is approx. 6.25 mg.

**Figure 17:**
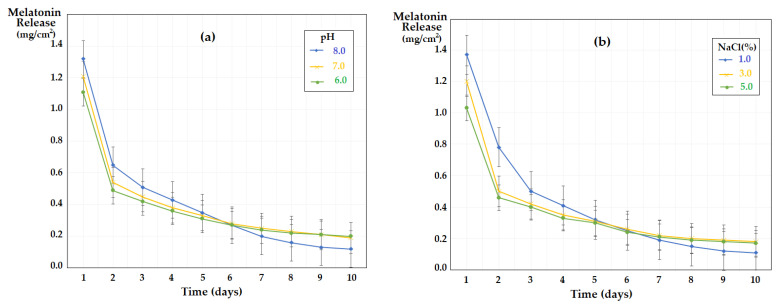
The variation of the amount of melatonin delivered daily per surface unit (cm^2^) of melatonin-chitosan-sulfonated ethylene-propylene-diene terpolymer composite membranes (Mel-Chi-sEPDM, M3), over a period of 10 days: (**a**) depending on the pH and (**b**) the salinity of the receiving phase.

The results obtained (Figure 17) show that the release rate of melatonin starts with approx. 1.25 ± 0.15 mg/cm^2^ per day, for the first day, and decreases constantly, reaching 0.1 ± 0.05 mg/cm^2^·per day, on the tenth day. In this case as well, the increase in pH slightly favours the release of melatonin (Figure 17a), and the increase in salinity causes a slower release of this hormone (Figure 17b).

What must be emphasized, from the perspective of the applications, is that after the fifth day, the amount of melatonin delivered drops almost five times, after which the level remains low and relatively constant.

These observations are useful for the administration of melatonin, by means of this type of composite membrane, in areas of the body with a higher pH, or in narrow places with profuse perspiration, but taking it to refresh the melatonin content of the membrane or to change the whole membrane after 5 days of application.

### 3.3. Aspects Regarding the Administration Method of Melatonin Using the Principle of the Studied Systems

The delivery of melatonin orally, in doses of 1 mg to 10.0 mg per day, is widespread and involves ingestion as pure powder or conditioned (formulated) powder using various compounds that ensure delayed release, in the form of cachets, capsules, or tablets [77]. Although other techniques and delivery procedures have been developed (gels, bandages, creams, patches) [78], the approach to the direction of release or delivery with membranes is of special interest, especially for cases of accidents resulting in scratches, punctures, tears, trauma, fractures, injuries caused by practicing various contact sports, the use of sports equipment, or motorized sports [79].

**Figure 18:**
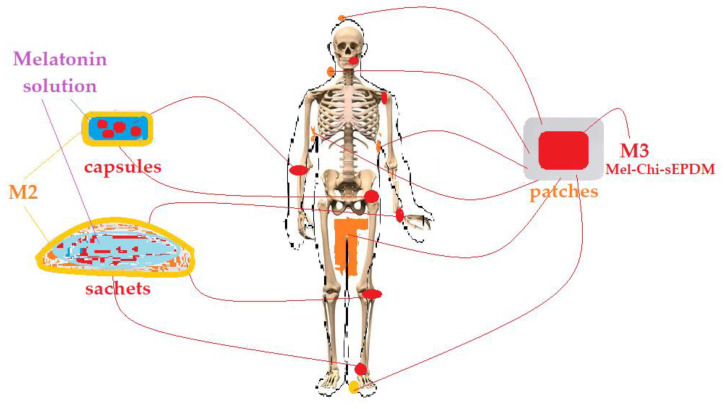
Possible ways to deliver melatonin using chitosan–sEPDM (M2) or melatonin–chitosan–sEPDM (M3) composite membranes.

In the case of the present study, the development of composite membranes, for the transport and delivery of melatonin, based on an ion polymer (sEPDM) with excellent mechanical, thermal, and chemical (pH, oxidative potential, bacterial attack) properties and a biopolymer (chitosan) that is biocompatible and interacts excellently with melatonin, ensures the diversification of delivery forms of this hormone in the case of sports biomedicine applications (Figure 18).

Thus, the M2 composite membrane, which allows the transport and delivery of melatonin from a quasi-saturated source phase toward the affected tissue, can be applied in the case of sprains, dislocations, joint fractures, broken or displaced teeth, as soft bags or soft capsules that, at certain intervals, can be “refuelled” without removing them from the place where they were placed (Figure 18).

For cases of superficial injuries (skin, head, fingers, spine), the composite membrane M3 is indicated to be applied as patches or adhesive dressings, the most suitable places being the narrow areas of the body.

Both types of membranes are included in the non-resorbable category, being removed after the lesions have either improved or healed.

Unlike oral administration, when melatonin comes into contact with a strongly acidic (pH 2–3, in the stomach) or basic (intestines) environment, in the case of the skin and oral cavity, the pH is close to neutral but highly dependent on the external contact environment, which involves studying transport and delivery over the entire pH scale.

## 4. Conclusions

This paper presents the preparation and characterization of chitosan and sulfonated ethylene-propylene-diene terpolymer (Chi-sEPDM) composite membrane, and melatonin-chitosan-sulfonated ethylene-propylene-diene terpolymer composite membranes (Mel-Chi-sEPDM), and its controlled release performance in synthetic aqueous solutions.

The membranes were obtained from an 8% sEPDM solution in toluene (*w/w*), in which chitosan or melatonin–chitosan (1:1, *w/w*) powders were dispersed in an ultrasonic field (sEPDM:Chi=1:1, *w/w* and Mel:Chi:sEPDM=0.5:0.5:1, *w/w/w*), through controlled vacuum evaporation. They were morphologically and structurally characterized by scanning electron microscopy (SEM), Fourier transform infrared spectroscopy (FTIR), energy-dispersive spectroscopy analysis (EDAX), thermal analysis (TG, DSC), thermal analysis coupled with infrared chromatography and analysis, and contact angle measurements, but also from the perspective of performance in the processes of transport and release of melatonin in dedicated environments (aqueous solution with controlled pH and salinity).

The transport of melatonin in the system with the receiving phase of variable pH is mainly determined by the difference in concentration between the aqueous phases, so by the solubility of melatonin in aqueous phases of relatively close composition, while the transport of melatonin to the receiving phase of controlled salinity corresponds to a coupled transfer mechanism in which the transport of melatonin from the source phase to the receptor phase is coupled with the transport of sodium ions in the opposite direction.

The results obtained for the release of melatonin show that chitosan-sulfonated ethylene-propylene-diene terpolymer (Chi-sEPDM) membrane (M2) allows a faster transfer and in a quantity that approaches the data from the literature, while sulfonated ethylene-propylene-diene terpolymer (sEPDM) membrane (M1) has a low but relatively constant release over time.

The prepared membranes can release melatonin in amounts between 0.4 mg/cm^2^·per day (M1), 1.6 mg/cm^2^·per day (M2), and 1.25 mg/cm^2^·per day (M3).

## Figures and Tables

**Table 1:** The characteristics of the used organic compounds.

Organic Compounds	Name and Symbol	Molar Mass (g/mol)	Solubility in Water (g/L)	pKa
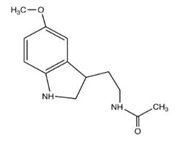	Melatonin (Mel)	232.28	2g/L; max. 3·10^−3^ mol/L	5.7 and 10.2
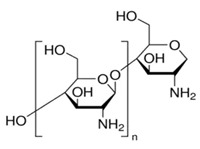	Chitosan (Chi)	1526.5	soluble in acid media (0.5 M HCl: 50 mg/mL)	6.2 to 7.0
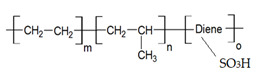	sulfonated ethylene-propylene-diene terpolymer (sEPDM)	2500–6500	soluble in toluene	1.9 to 2.2

**Table 2:** The macroscopic characteristics for the obtained membranes.

Material	Membrane Symbols	Thickness (µm)	Membrane View (Photo)	Low Magnitude SEM (Membrane Surface)	Contact Angle (θ°)
sEPDM	M1	50 ± 2	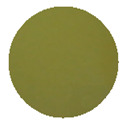	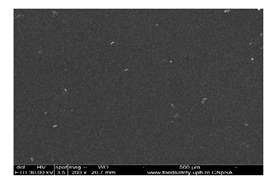	73 ± 3
Chi-sEPDM	M2	51 ± 4	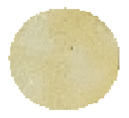	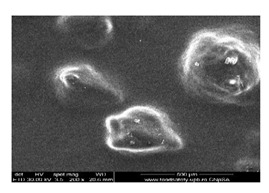	42 ± 5
Mel-Chi-sEPDM	M3	49 ± 5	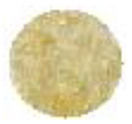	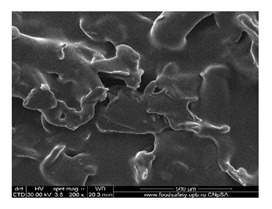	33 ± 5

**Table 3:** Energy dispersive spectroscopy analysis (EDAX) for the prepared membranes.

Membranes	M1			M2			M3		
Surface Composition	Weight (%)	Atomic (%)	Error (%)	Weight (%)	Atomic (%)	Error (%)	Weight (%)	Atomic (%)	Error (%)
**C** K	94.48	95.88	3.07	96.37	97.3	2.29	93.16	94.92	2.88
**O** K	5.28	4.02	29.5	3.49	2.64	30.79	6.44	4.93	20.94
**S** K	0.24	0.09	62.31	0.15	0.06	61.64	0.4	0.15	16.27

**Table 4:** The FTIR 2D maps for sEPDM membrane (M1) and composite membrane (M2 and M3).

	3345 cm^−1^	1385 cm^−1^	1050 cm^−1^	728 cm^−1^
M1	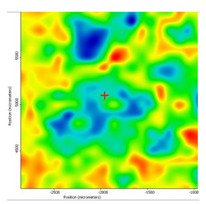	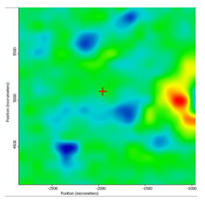	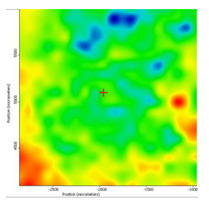	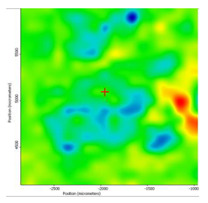
M2	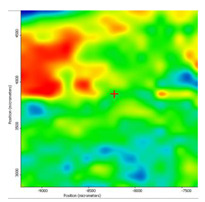	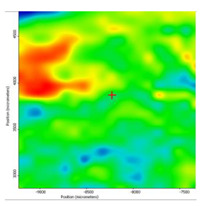	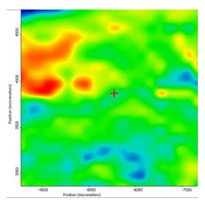	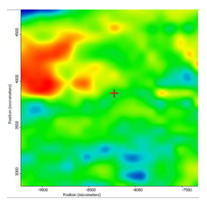
MM3	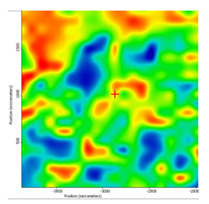	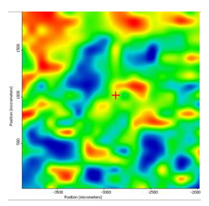	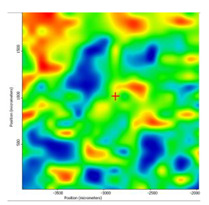	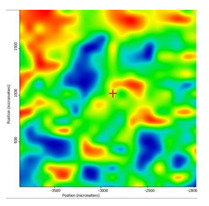

## Data Availability

Data are contained within the article.

## Supplementary Materials

The following are available online at https://www.mdpi.com/article/10.3390/membranes13030282/s1, Figure S1: Schematic presentation of the melatonin biomedical implication, Figure S2: Trace for evolving SO_2_ (1367 cm^−1^) vs. temperature (**a**); and trace for evolving CO_2_ (2355 cm^−1^) vs. temperature (**b**), Figure S3: Trace for evolving **(a)** SO_2_ (1367 cm^−1^); (**b**) CO_2_ (2355 cm^−1^); (**c**) hydrocarbons (2964 cm^−1^); and (**d**) H_2_O (3566 cm^−1^) vs. temperature, Figure S4: Trace for evolving (**a**) SO_2_ (1367 cm^−1^); (**b**) CO_2_ (2355 cm^−1^); (**c**) hydrocarbons (2964 cm^−1^); and (**d**) H_2_O (3566 cm^−1^) vs temperature.
