# Supplementary material for: Chitosan–sEPDM and Melatonin–Chitosan–sEPDM Composite Membranes for Melatonin Transport and Release
Source: Membranes (Basel). 2023 Feb 27;13(3):282. doi: 10.3390/membranes13030282 (PMC10057635; doi:10.3390/membranes13030282)
Supplement: Supplementary file 1 [file membranes-13-00282-s001.zip › membranes-2252259-supplementary.pdf]

Article

Chitosan–sEPDM and Melatonin–Chitosan–sEPDM composite membranes for melatonin transport and release

Florentina Mihaela Păncescu [1], Abbas Abdul Kadhim Klaif Rikabi [1,2], Ovidiu Cristian Oprea [3], Alexandra Raluca Grosu [1], Aurelia Cristina Nechifor [1], Vlad-Alexandru Grosu [4,*], Szidonia-Katalin Tanczos [5], Florina Dumitru [3], Gheorghe Nechifor [1,*] and Simona Gabriela Bungău [6]

[1] Analytical Chemistry and Environmental Engineering Department, University Politehnica of Bucharest, Bucharest 011061, Romania; florynicorici@yahoo.com (F.M.P.); dr.abbas.rikabi@atu.edu.iq (A.A.K.K.R.); andra.grosu@upb.ro (A.R.G.); aureliacristinanechifor@gmail.com (A.C.N.); ghnechifor@gmail.com (G.N.)

[2] Al–Mussaib Technical College/Al–Furat Al–Awsat Technical University (ATU), Babylon - Najaf Street, Kufa 54003, Iraq; dr.abbas.rikabi@atu.edu.iq; (A.A.K.K.R.)

[3] Department of Inorganic Chemistry, Physical Chemistry and Electrochemistry, University Politehnica of Bucharest, Bucharest 011061, Romania; ovidiu.oprea@upb.ro (O.C.O.); d_florina@yahoo.com (F.D.)

[4] Department of Electronic Technology and Reliability, Faculty of Electronics, Telecommunications and Information Technology, University Politehnica of Bucharest, Bucharest 061071, Romania; vlad.grosu@upb.ro (V.-A.G)

[5] Department of Bioengineering, University Sapientia of Miercurea-Ciuc, Miercurea-Ciuc 500104, Romania; tczszidonia@yahoo.com (S.-K.T.)

[6] Department of Pharmacy, Faculty of Medicine and Pharmacy, University of Oradea, Oradea 410028, Romania; sbungau@uoradea.ro (S.G.B.)

* Corresponding authors: vlad.grosu@upb.ro (V.-A.G); ghnechifor@gmail.com (G.N.)

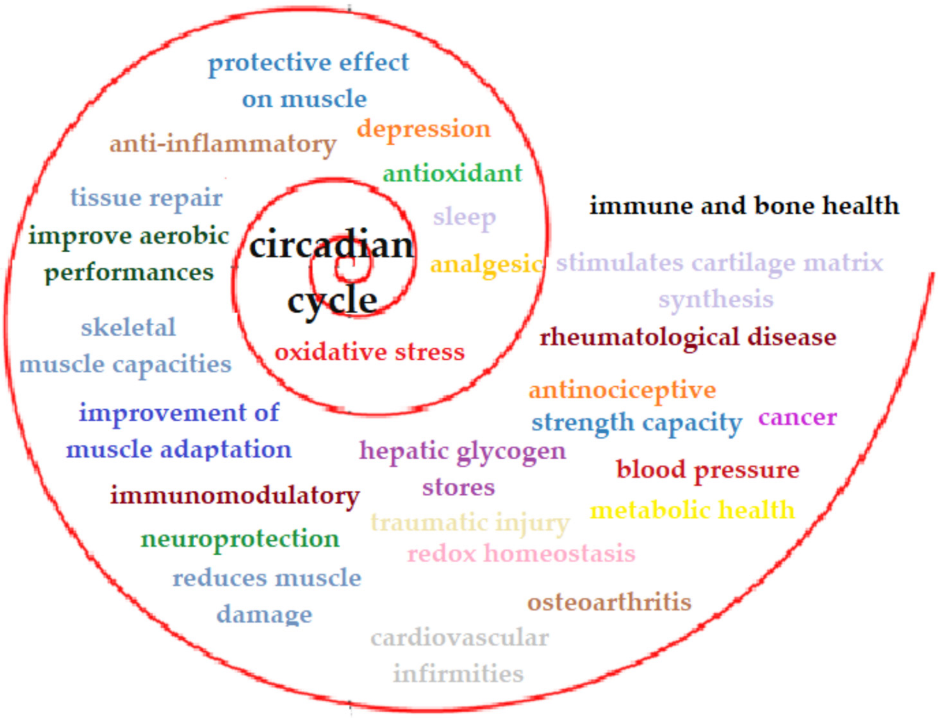

Figure S1. Schematic presentation of the melatonin biomedical implication.

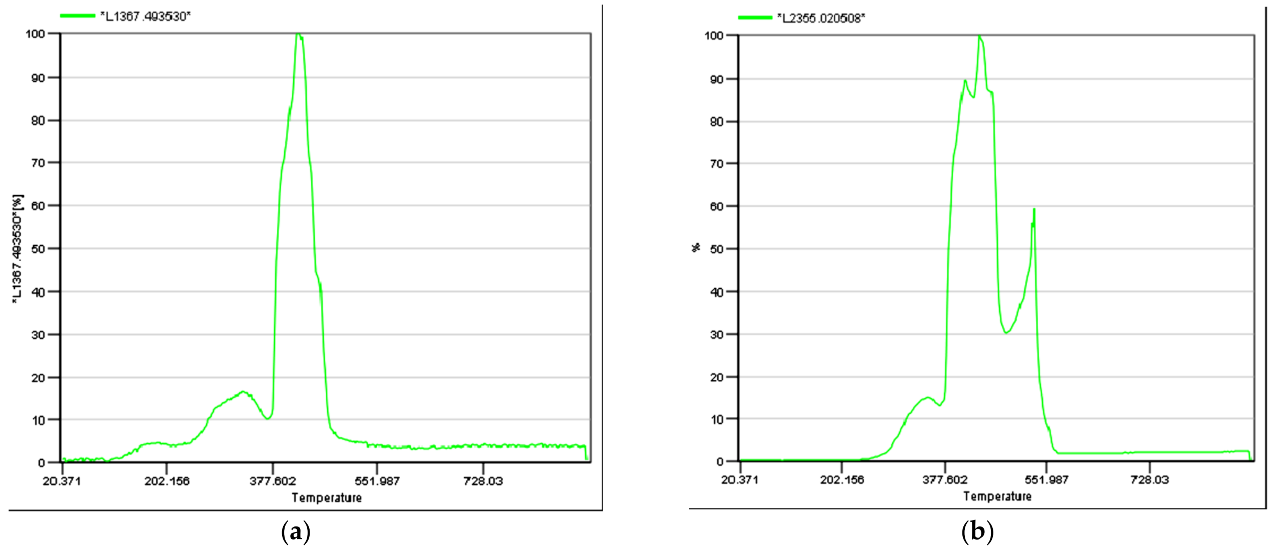

Figure S2. Trace for evolving SO_2 (1367 cm^{-1}) vs temperature (**a**); and trace for evolving CO_2 (2355 cm^{-1}) vs temperature (**b**).

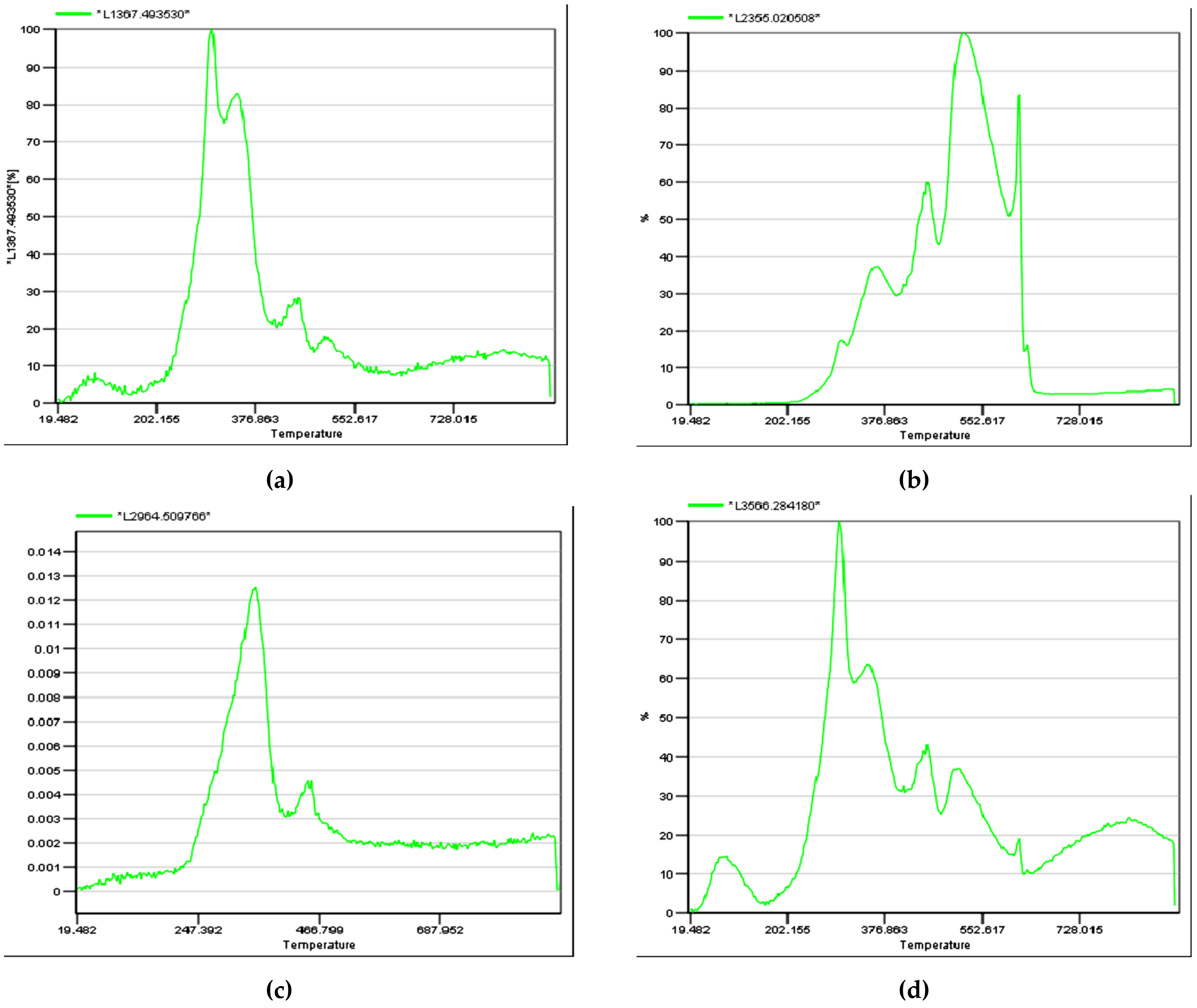

Figure S3. Trace for evolving (**a**) SO_2 (1367 cm^{-1}); (**b**) CO_2 (2355 cm^{-1}); (**c**) hydrocarbons (2964 cm^{-1}); and (**d**) H_2O (3566 cm^{-1}) vs temperature.

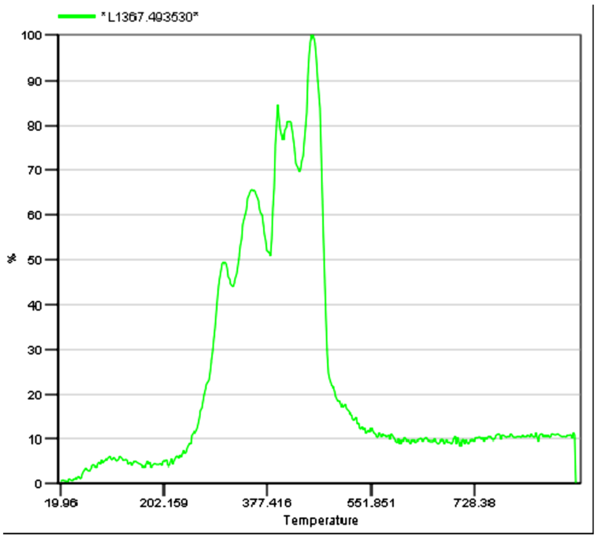

(a)

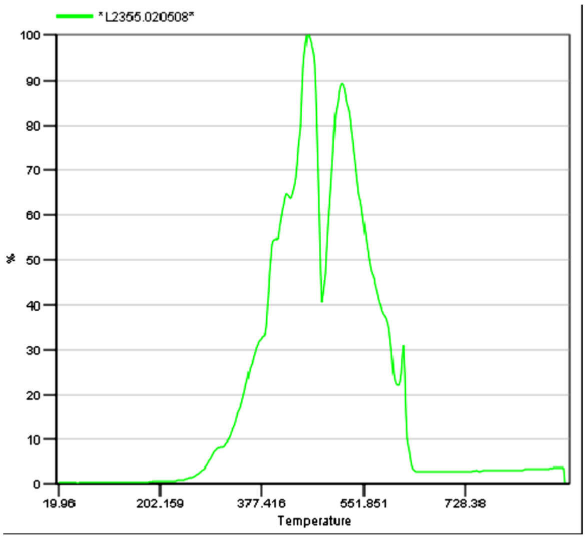

(b)

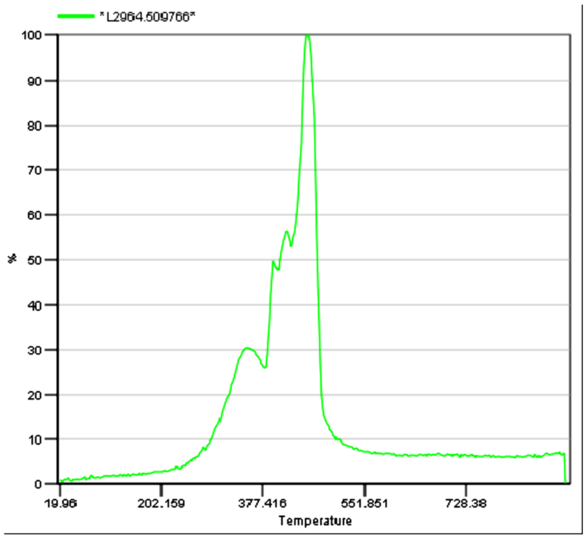

(c)

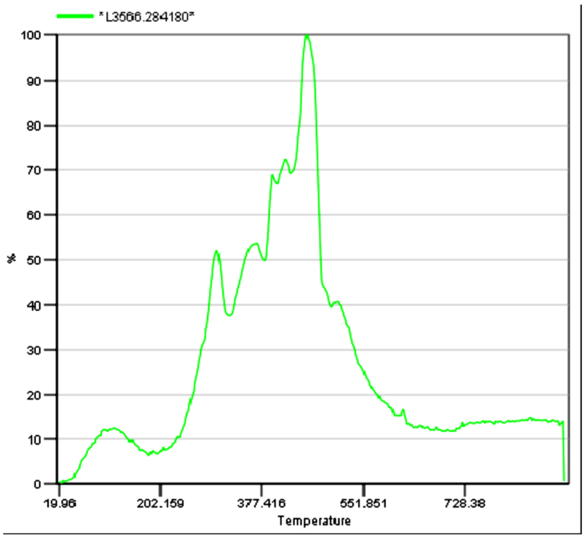

(d)

Figure S4. Trace for evolving (**a**) SO_2 (1367 cm^{-1}); (**b**) CO_2 (2355 cm^{-1}); (**c**) hydrocarbons (2964 cm^{-1}); and (**d**) H_2O (3566 cm^{-1}) vs temperature.
